# New frontiers in salivary extracellular vesicles: transforming diagnostics, monitoring, and therapeutics in oral and systemic diseases

**DOI:** 10.1186/s12951-024-02443-2

**Published:** 2024-04-12

**Authors:** Li Cui, Jiarong Zheng, Ye Lu, Pei Lin, Yunfan Lin, Yucheng Zheng, Rongwei Xu, Zizhao Mai, Bing Guo, Xinyuan Zhao

**Affiliations:** 1 Stomatological Hospital, School of Stomatology, Southern Medical University, Guangzhou, Guangdong 510280 China; 2 Department of Dentistry, The First Affiliated Hospital, Sun Yat-Sen University, Guangzhou, 510080 China

**Keywords:** Salivary extracellular vesicle, Early detection, Therapeutic potential, Disease monitoring

## Abstract

Salivary extracellular vesicles (EVs) have emerged as key tools for non-invasive diagnostics, playing a crucial role in the early detection and monitoring of diseases. These EVs surpass whole saliva in biomarker detection due to their enhanced stability, which minimizes contamination and enzymatic degradation. The review comprehensively discusses methods for isolating, enriching, quantifying, and characterizing salivary EVs. It highlights their importance as biomarkers in oral diseases like periodontitis and oral cancer, and underscores their potential in monitoring systemic conditions. Furthermore, the review explores the therapeutic possibilities of salivary EVs, particularly in personalized medicine through engineered EVs for targeted drug delivery. The discussion also covers the current challenges and future prospects in the field, emphasizing the potential of salivary EVs in advancing clinical practice and disease management.

## Background

Biopsy of living tissue remains the gold standard for disease diagnosis. Yet, as an invasive technique, it is hindered by limitations such as restricted sample range, the risk of complications, and its static nature, which may not capture the disease’s dynamics [1]. Over the past decade, advancements in the study of circulating tumor cells [2], tumor-derived DNA [3], and exosomes [4, 5] have paved the way for liquid biopsy to emerge as a superior diagnostic approach. Saliva, in particular, offers a non-invasive and convenient alternative for biopsy, presenting significant promise for its integration into precision medicine.

Saliva is a vital component of human body fluids, acting as a repository for an array of biomarkers, including metabolic products, DNA, RNA, proteins, and microbial populations [6–8]. It mirrors the blood’s composition due to physiological exchange mechanisms between the salivary glands and the systemic circulation. Saliva’s role in clinical diagnostics is underscored by its non-invasive collection methods, which offer advantages over blood draws by eliminating the risk of cross-infection, reducing costs, and increasing patient compliance due to its minimally intrusive nature [9, 10]. Furthermore, saliva collection is not constrained by the location or time, facilitating immediate and flexible disease diagnostics, which is particularly advantageous over conventional tissue biopsies. These attributes make saliva an exceptionally promising medium for biomarker research and its translation into clinical practice.

Transitioning from the broader concept of saliva’s role in non-invasive diagnostics, we focus on the specific entities within saliva that could hold the key to unlocking a new era of disease detection and monitoring: salivary extracellular vesicles (EVs), including exosomes. Enclosed by a lipid bilayer, salivary EVs are crucial for cellular communication and carry biomolecules that mirror the health status of patients [11, 12]. Their analysis could enhance liquid biopsy techniques by providing a stable, non-invasive means to detect and monitor diseases, contributing to advances in precision medicine.

While a few review papers have focused on the clinical significance of salivary EVs, the novelty of our review should be noted for several reasons [13, 14]. Firstly, we have systematically summarized new methods for the isolation and enrichment of salivary EVs, offering methodological choices for future research. Secondly, there has been an abundance of recent studies utilizing salivary EVs for the diagnosis and monitoring of disease activity; our review provides a timely and objective synthesis of the latest findings. Thirdly, we discuss the potential and novel perspectives of engineering salivary EVs for therapeutic applications. Lastly, we highlight the emerging challenges and propose novel considerations for the clinical application of salivary EVs.

### Overview of EV biology

EVs are primarily classified into three types: apoptotic bodies, microvesicles, and exosomes [15]. These subtypes of EVs are distinguished by their size and biogenesis. A challenge in this field is the absence of specific markers for each of these EV subpopulations. Apoptotic bodies are the largest, typically ranging from 500 to 5000 nm in diameter. They form during apoptosis and contain a variety of cellular components, playing a key role in the removal of dying cells and impacting immune responses [16]. Microvesicles, also known as ectosomes, are medium in size, ranging from approximately 100 to 1000 nm. They are produced by the outward budding and fission of the plasma membrane, differing from apoptotic bodies in size, formation process, content, and specific membrane antigens [17, 18]. Exosomes are the smallest EVs, measuring between 30 and 150 nm in diameter. They are generated within the endosomal network and released upon the fusion of multivesicular bodies (MVBs) with the plasma membrane [19]. MVBs selectively package cellular components through various sorting mechanisms [20]. Exosomes are particularly rich in membrane-associated proteins, such as CD63 and CD81 [21]. The secretion process relies on the RAB family of GTP-binding proteins for vesicle transport and the SNARE family for lipid bilayer fusion [22]. Not all MVBs are destined to release exosomes; some are directed to lysosomes for content degradation under certain conditions [23].

EVs are secreted by a diverse range of cell types, including immune cells, neurons, and tumor cells, and are present in various bodily fluids such as blood, urine, and saliva [23–25]. Their complex structure harbors an array of bioactive molecules, including nucleic acids, proteins, and metabolites, which reflect the state of their cells of origin [26–28]. Consequently, EVs present in biofluids offer an accurate representation of cellular and systemic health and are invaluable for the early detection and thorough monitoring of a variety of health conditions. Salivary EVs originate from a variety of cellular sources within and adjacent to the oral cavity. Primarily, they are secreted by the cells of salivary glands, including the parotid, submandibular, and sublingual glands. Additionally, a significant contribution comes from various cell types in the oral cavity, such as oral epithelial cells, immune cells, and neuronal cells, each adding unique biomolecular signatures to the EVs. The oral microbiome further enriches this profile by releasing its own set of EVs [29]. Beyond the local environment, systemic influences are evident, with blood-derived EVs entering the saliva through the salivary gland microvasculature, and in certain conditions, such as gastroesophageal reflux disease, exosomes from the esophagus and stomach may also be detected. This complex and integrated origin of salivary EVs underlines their potential as comprehensive indicators for both oral and systemic health assessments.

### Advantages for salivary EVs compared to whole saliva

Salivary EVs offer significant advantages over whole saliva by enhancing biomarker detection, ensuring sample integrity, and allowing precise disease monitoring. Firstly, whole saliva samples are highly susceptible to contamination from external substances. During saliva formation and its expulsion from the oral cavity, contaminants such as food debris and oral bacteria can easily mix in, negatively impacting subsequent analyses [13]. Additionally, salivary glands secrete enzymes such as amylase into the saliva. The presence of these contaminants and enzymatic proteins, particularly, can severely interfere with and obscure the detection of low-abundance protein biomarkers in whole saliva. However, the process of extracting and concentrating salivary EVs is designed to separate them from contaminants, thereby reducing sample complexity. This allows for the detection of low-abundance proteins in whole saliva, significantly improving the sensitivity and specificity of the detection.

Secondly, although saliva contains components other than EVs, such as shed oral mucosal cells, DNA/RNA molecules, and cytokines, the proteins and RNA in saliva can degrade and denature quickly due to the presence of a high concentration of enzymes once they leave their optimal environment, leading to insufficient detection sensitivity and specificity, and reducing the accuracy and efficiency of the examination [30]. In contrast, salivary EVs have a lipid bilayer protective structure that prevents the degradation of their contents by exogenous enzymes, thereby better preserving the integrity and stability of the sample [31]. Moreover, salivary EVs act as a barrier to low-molecular-weight substances. The intact bilipid membrane of EVs selectively restricts the entry of hydrophilic molecules [32], potentially enhancing the specificity and accuracy of disease detection using these vesicles.

**Fig. 1:**
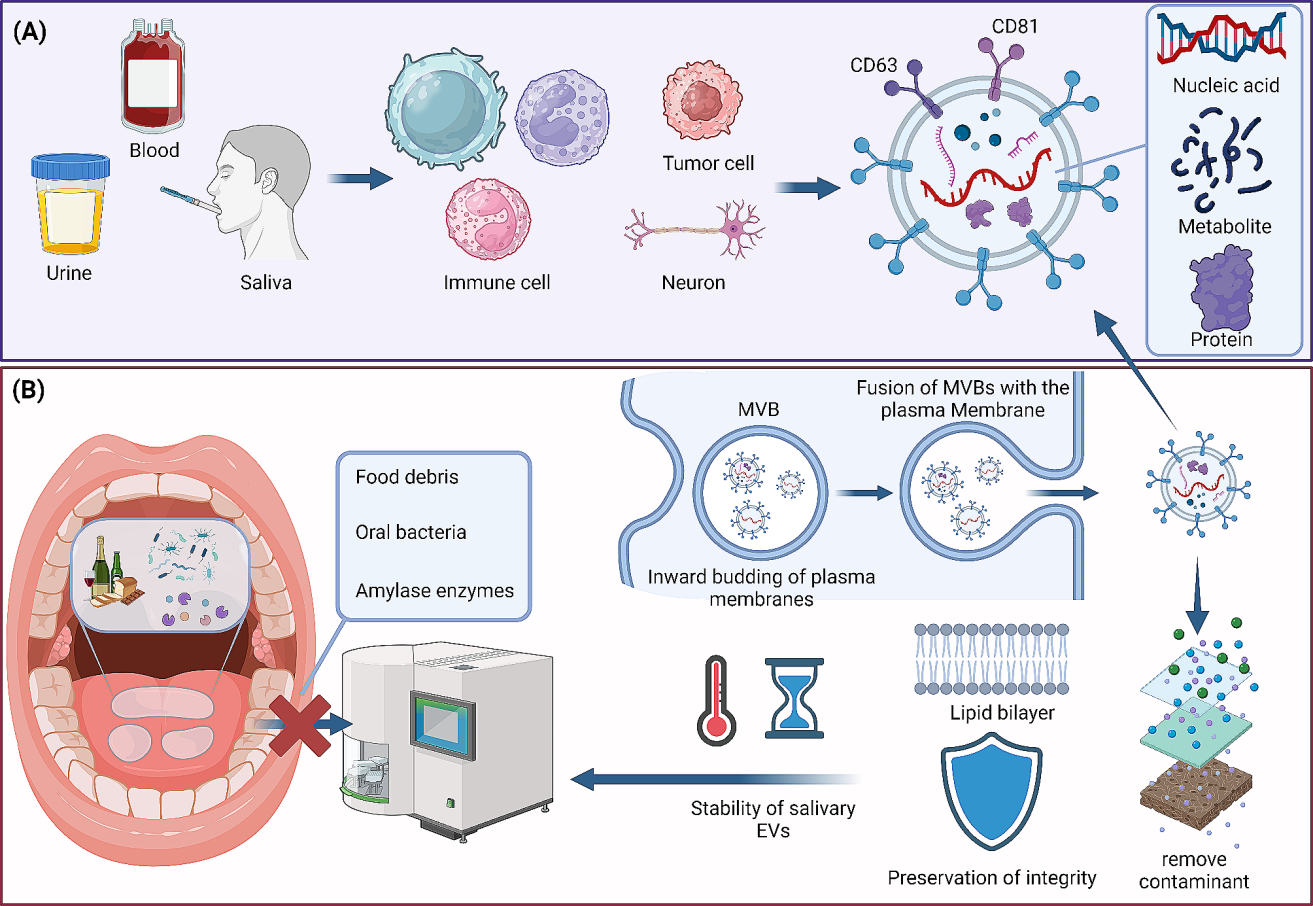
EV biogenesis and the advantages of salivary EVs for disease detection and monitoring. EV biogenesis and the advantages of salivary EVs for disease detection and monitoring. (**A**) Salivary EVs, secreted by various types of cells, can be detected in bodily fluids. (**B**) Compared to whole saliva, salivary EVs offer notable advantages, including effective removal of contaminants, maintenance of integrity, and enrichment for targets. These comprehensive properties establish salivary EVs as a key component in biomarker detection

Finally, salivary EVs can enrich components from specific cellular origins, allowing for targeted detection of key changes in diseased tissues, greatly enhancing the quantitative detection accuracy for specific proteins or RNA biomarkers. For instance, tumor-specific proteins and RNA can be obtained from tumor cell-derived salivary EVs. Monitoring EVs derived from epithelial cells can help in the surveillance of oral mucosal lesions, among other things. Furthermore, salivary exosomes exhibit remarkable long-term stability, maintaining membrane integrity for up to 20 months at 4 °C [14, 33]. Key exosomal markers such as DPP IV and Alix remained intact, and the exosomes proved resilient to treatments like detergents and freeze-thaw cycles, highlighting their potential for clinical use [34] (Fig. 1).

### The conventional methodologies for isolating salivary EVs

**Fig. 2:**
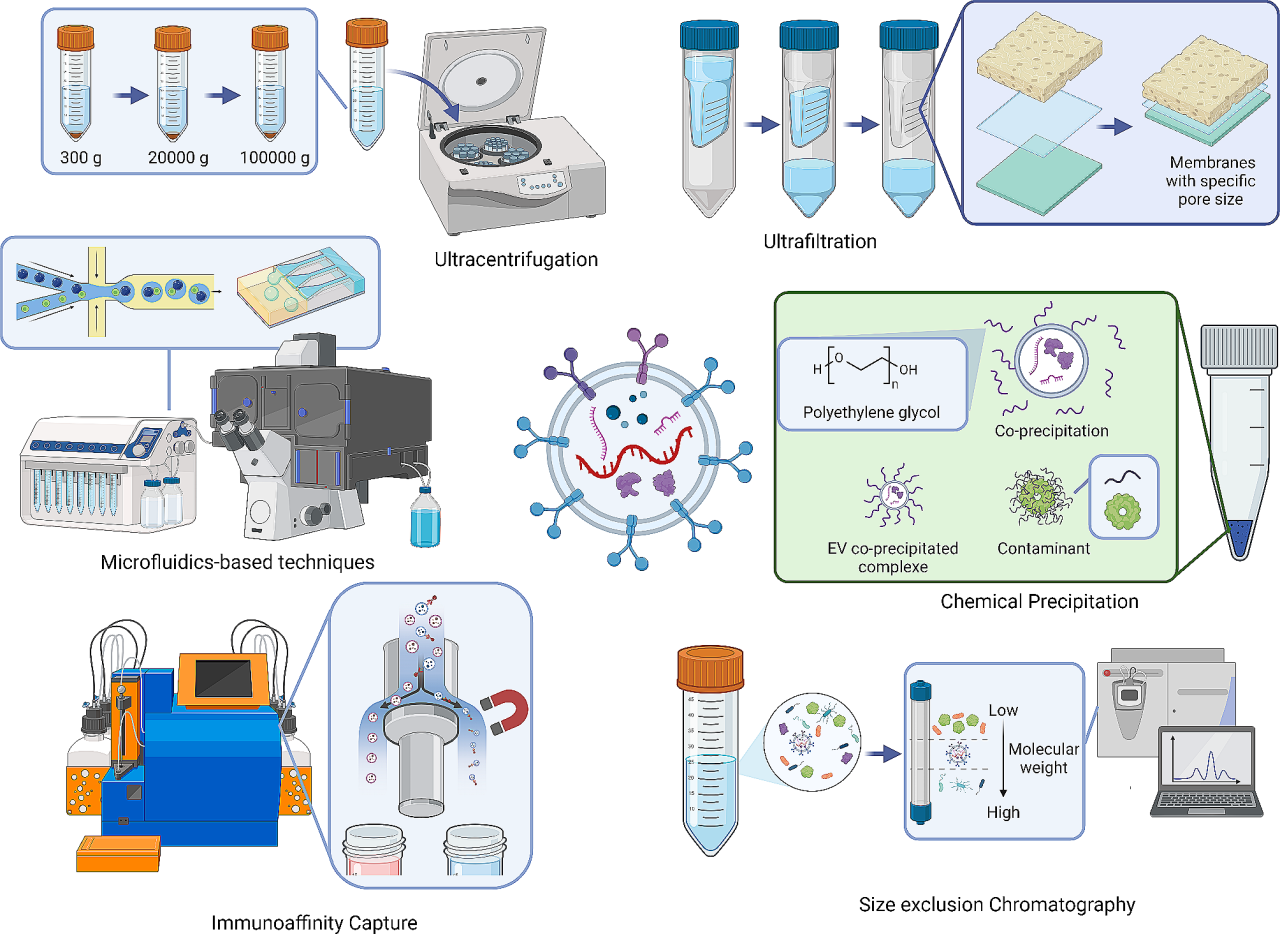
The conventional isolation method for salivary EVs. Conventional methodologies for isolating salivary EVs encompass a range of techniques, including ultracentrifugation, ultrafiltration, chemical precipitation, size exclusion chromatography, immunoaffinity capture, and microfluidics

#### Ultracentrifugation

Ultracentrifugation, a key technique for isolating EVs from saliva, includes differential and density gradient methods. Ultracentrifugation-isolated salivary EVs showed a higher expression of specific markers, were monodispersed, and teacup-shaped, while polyethylene glycol (PEG) resulted in irregular shapes and a broader size range. Proteomic analysis revealed ultracentrifugation as superior for isolating EV-related proteins [35]. Importantly, density gradient ultracentrifugation yielded higher quantities and purity of salivary EVs compared to size-exclusion chromatography, ultracentrifugation, and ultracentrifugation plus filtration [34]. Interestingly, using equilibrium density-gradient centrifugation with extended 96-hour ultracentrifugation, saliva-derived EVs from healthy individuals showed distinct buoyancy drifts, identifying subclasses with classical exosomal markers and those linked to membrane remodeling and vesicle trafficking [36].

#### Ultrafiltration

Ultrafiltration, a common size-based separation technique, uses membranes with specific pore sizes to concentrate EVs from large volumes, like cell culture media. This method allows smaller molecules or EVs to pass through, retaining larger particles. Known for processing multiple samples simultaneously, ultrafiltration saves time but faces challenges such as membrane clogging and protein contamination. To address these, it’s often combined with other methodologies. For instance, ultrafiltration and size-exclusion chromatography have been used to isolate human salivary exosomes, revealing diverse RNA compositions through next-generation sequencing. Similarly, salivary EVs can be isolated effectively using ultrafiltration with chemical precipitation [37].

#### Chemical precipitation method

Chemical precipitation methods, particularly PEG precipitation, are employed to isolate EVs based on their interactions with hydrophilic polymers. PEG interacts with hydrophobic protein and lipid molecules of EVs, leading to co-precipitation [38]. Although PEG precipitation can be costly and has challenges in avoiding protein aggregate contamination, its simplicity and effectiveness have led to its widespread use in commercial kits like ExoQuick and Total Exosome Isolation. When applied to saliva samples, these kits can efficiently isolate intact EVs, yielding high amounts of EVs suitable for downstream analyses. Notably, Total Exosome Isolation Solution resulted in the highest protein concentrations in salivary EVs when compared to several other techniques, including ultracentrifugation, ExoGAG, and ExoQuick [38].

#### Size exclusion chromatography (SEC)

SEC effectively isolates salivary EVs, separating molecules by size as smaller ones penetrate the porous beads in the column, while larger EVs elute earlier. This gentle method preserves EV integrity and minimizes protein contamination, making it suitable for functional studies and biomarker discovery. However, a drawback of this method is that it can dilute the sample, which may require additional steps to concentrate the sample afterward. Despite these limitations, SEC yielded a higher quantity of salivary EVs than ultracentrifugation, with no notable differences in dimensions between the two methods. Moreover, Izon’s qEVOriginal-70 nm columns yielded the highest purity in salivary EV isolation when compared to various other methods, indicating superior efficacy in separating EVs from other similarly sized particles [38].

#### Immunoaffinity capture

Immunocapture techniques for EV isolation utilize antibodies specific to EV membrane proteins. This method involves capturing EVs with magnetic beads coated with targeted antibodies, followed by magnetic separation. It enables the isolation of intact, high-purity EVs while preserving their biological activity, making them suitable for experimental applications like cell co-culture and in vivo studies. Additionally, immunocapture can be customized to isolate EVs from certain cell types or disease states, especially cancer, by targeting tumor-specific biomarkers. This specificity renders immunocapture a rapid and clinically relevant method for isolating EVs from saliva samples.

#### Microfluidics-based techniques

Microfluidics-based techniques for isolating EVs represent a significant advancement in EV research. These methods utilize small-scale fluidic channels to manipulate and isolate EVs with high precision. Various microfluidic devices have been designed for EV isolation, offering the dual benefits of high-purity separation and the ability to detect specific EV subpopulations in small volumes of body fluid with high throughput. For instance, microfluidic devices that employ dielectrophoretic (DEP) forces have shown great promise in the isolation of EVs. These devices apply non-uniform electric fields to selectively separate exosomes based on their polarizability. This integration of DEP in microfluidics allows for precise, efficient, and less invasive EV isolation, enhancing purity and yield while reducing processing time [39]. For instance, a novel insulator-based DEP microfluidic device with a borosilicate micropipette array efficiently isolates exosomes from biofluids like plasma, serum, and saliva. Processing 200 µL samples in just 20 min at low voltage, this easily fabricated device provides a cost-effective, high-yield, and pure extraction method, enhancing biomarker research and diagnostics [40]. Deterministic lateral displacement (DLD) technology, a microfluidic method for size-based passive particle separation, excels in EV isolation with its ability to alter the flow paths of larger particles while sparing smaller ones. This feature renders DLD ideal for exosome separation and purification. The device, comprising an array of cylindrical structures tailored to specific sizes, allows for efficient EV isolation from larger cells, catering to high-throughput screening needs. DLD’s label-free process significantly reduces sample contamination, enhancing both separation efficiency and sample purity, vital for biomedical research and clinical applications. Despite its advantages, DLD faces challenges like low throughput, pillar clogging, and cumbersome setup, with current limitations in size-based separation impacting EV saturation and recovery [41]. Notably, viscoelastic flow-based microfluidic devices exploit the unique properties of viscoelastic fluids to separate EVs from other cellular components in a biological sample. As the sample flows through a microchannel filled with a viscoelastic medium, exosomes experience different flow-induced forces compared to larger particles, leading to their effective separation. This method is particularly advantageous as it enables gentle, label-free, and low-shear isolation of EVs, preserving their integrity while ensuring efficient separation [42]. Similarly, acoustic-wave-based microfluidic devices have emerged as a highly efficient method for EV isolation. Utilizing acoustic waves to generate pressure differences within microfluidic channels, these devices can separate EVs from other cellular components based on size and density. This technique is gentle, preserving the biological and structural integrity of the EVs. Its label-free, non-invasive nature allows for high-throughput and high-purity isolation [43]. For instance, the acoustofluidic platform, fusing acoustics and microfluidics, effectively isolates salivary exosomes, outperforming differential centrifugation by yielding 15 times more exosomal small RNA. This method shows promise for high-purity and high-yield extraction of salivary exosomes, optimizing HPV detection in liquid biopsy applications [44]. Moreover, immunoaffinity capture-based microfluidic devices utilize surface-bound antibodies that selectively bind to certain proteins present on the EVs’ surface. This method ensures highly specific and targeted isolation of EVs, particularly useful for isolating disease-specific or subtype-specific EVs. The integration of immunoaffinity principles into microfluidic systems facilitates a streamlined, efficient process, enhancing the purity and relevance of the isolated EVs (Fig. 2).

### Novel methodologies for enriching, quantifying and identifying salivary EVs

The enrichment of salivary EVs is crucial for the early detection and monitoring of various medical conditions. By concentrating these vesicles from saliva, it becomes possible to enhance the accuracy and sensitivity of diagnostic assays, enabling the detection of disease markers that are often present in only trace amounts in the early stages of a condition. Improved enrichment techniques are key to unlocking their full potential in personalized medicine and clinical research. Recently, a novel strategy employs Fe_3_O_4_@SiO_2_-aptamer nanoparticles to capture and concentrate lung cancer exosomes, with duplex-specific nuclease serving as an amplification tool to enhance miRNA signal detection. This method shows promising potential for lung cancer diagnosis by enabling the sensitive and stable detection of exosomal miR-205 in both saliva and urine samples, with a sensitivity reaching as low as 7.76 pM [45].

Accurately quantifying salivary EVs is of great importance for advancing both diagnostic and therapeutic applications. This precise quantification is not just a technical achievement; it represents a fundamental key to unlocking a deeper understanding of the role of EVs in various physiological and pathological states. By accurately measuring EV concentrations in saliva, we can establish more definitive correlations between EV profiles and specific health conditions. This enhances diagnostic precision in the early detection of diseases, in monitoring disease progression, and in evaluating responses to treatments. Recently, a novel fluorescent biosensor was developed for the one-step, sensitive quantification of salivary exosomes using magnetic and fluorescent bio-probes. This biosensor combines DNA concatamers, quantum dots, and aptamers on magnetic microspheres for efficient exosome capture, enabling a “one exosome-numerous QDs” amplification effect. It offers rapid (0.5 h) and highly sensitive quantification, characterized by a low limit of detection (500 particles/µL), a wide detection range spanning three orders of magnitude, and robust performance in complex samples, evidenced by its impressive quantitative capacity with an R-squared value of 0.998 [46]. Interestingly, a new hybrid capture bioassay for measuring microvesicle tissue factor (MVTF) activity in human body fluids has been introduced, bypassing traditional high-speed centrifugation methods. This assay integrates specific immunocapture of MVs using anti-CD29 and anti-CD59 coated magnetic beads with accurate TF activity measurement. Its application across diverse body fluids like plasma, pleural fluid, and saliva has demonstrated improved reproducibility and maintained sensitivity [47]. This development could have profound implications for the future of MV research, potentially leading to more precise diagnostic and therapeutic strategies in clinical settings.

Differentiating EVs from other nanocarriers is crucial for avoiding misinterpretation of results due to the presence of similar-sized particles, ensuring more accurate biomarker identification and paving the way for more targeted and effective treatments. A new continuous isoelectric fractionation technique has been developed for efficient separation of EVs, lipoproteins, and ribonucleoproteins from biofluids including saliva. This high-throughput method, utilizing a linear pH profile and machine learning for recalibration, achieves not only high purity and yield across various biofluids but also reaches a resolution of 0.3 ΔpI. This level of resolution is sufficient to separate different nanocarriers and their subclasses effectively [48]. Importantly, NanoFCM demonstrated superior performance in analyzing EVs ranging from 40 to 200 nm, effectively quantifying individual EV particles, identifying uncommon EV marker subsets, and facilitating the simultaneous localization of multiple surface markers. In contrast, Aurora was more effective in analyzing EVs larger than 200 nm and excelled in identifying EVs stained with various surface markers [49]. Similarly, He et al. presents a new electrochemical method for detecting salivary exosomes, using a red blood cell membrane engineered with CD63 aptamer on a gold electrode for targeted capture. This technique provides sensitive detection of target salivary exosomes across a broad linear range from 5 × 10² to 1 × 10^2^ particles per mL, coupled with a low detection limit of 2.07 × 10² particles per mL, showing significant potential for the clinical diagnosis of oral diseases using salivary exosomes [50]. The summary of novel methodologies for enriching, quantifying and identifying salivary EVs in the review is provided in Table 1.

**Table 1:** Novel methodologies for enriching, quantifying and identifying merged as key tools for salivary EVs

Method	Purpose	Sample	Time	Detection limit	Specificity/sensitivity/linearity	Ref
Fe_3_O_4_@SiO_2_-aptamer nanoparticles	Enriching exosomes	Saliva/urine	NA	7.76 pM	Sensitivity: high	[45]
Quantum dot-based platform	Quantifying exosomes	Saliva	30 min	500 particles/µL	Sensitivity: high linearity = 0.998	[46]
Hybrid capture bioassay	Measuring microvesicle tissue factor activity	Body fluids	NA	NA	Specificity: high; sensitivity: high linearity > 0.99	[47]
Nanocarrier fractionation platform	Identifying EVs	Body fluids	30 min	NA	Specificity: high sensitivity: high	[48]
NanoFCM	Identifying EVs	Body fluids	NA	NA	High sensitivity for detecting EVs ranging from 40–200 nm	[49]
Aurora	Identifying EVs	Body fluids	NA	NA	High sensitivity for detecting EVs > 200 nm	[49]
Red blood cell membrane engineered-based method	Identifying EVs	Saliva	NA	207 particles/mL	Specificity: high sensitivity: high	[50]

### Salivary EVs as biomarkers for oral disease diagnosis and monitoring

Salivary EVs hold significant potential for the detection and management of oral diseases, given their direct interaction with the oral environment. The anatomical proximity of salivary glands to the oral cavity ensures that salivary EVs are an abundant and rich source of biological markers that reflect local pathophysiological conditions. These vesicles encapsulate and transport molecular signatures of oral health status, including responses to infections, inflammation, and neoplastic transformations. Consequently, analyzing salivary EVs offers a non-invasive, real-time snapshot of oral tissue integrity, making them highly relevant for early diagnosis, monitoring disease progression, and evaluating treatment responses in oral health care.

#### Periodontitis

Periodontitis is an inflammatory condition precipitated by dental plaque and influenced by multiple factors that elicit an immune response in the host. This condition not only results in the deterioration of periodontal support structures but may also have systemic health implications [51]. Distinct microRNAs (miRNAs), proteins, and molecular patterns in salivary EVs effectively differentiate periodontitis patients from healthy individuals, underscoring their diagnostic potential. In a pilot study with 29 participants, three miRNAs, namely miR-140-5p, miR-146a-5p, and miR-628-5p, were markedly elevated in the salivary EVs of periodontitis patients [52]. Another study found that levels of miR-223-3p in salivary exosomes were lower in periodontitis patients compared to healthy individuals. This particular miRNA plays a crucial role in regulating inflammation in periodontitis by targeting the NLRP3 protein [53]. Moreover, in comparison to healthy saliva, exosomal miRNAs from chronic periodontitis samples were predominantly down-regulated, although miR-125a-3p was notably upregulated. Owing to its strong correlation with periodontal pocket depth, miR-125a-3p emerges as a potential key biomarker for chronic periodontitis [54]. Beyond salivary exosomal miRNAs, other components have also been reported to be dysregulated in the saliva samples from periodontitis patients. For instance, in young adults with severe periodontitis, salivary exosomes contained 26 proteins not detected in the healthy cohort. Conversely, the healthy group exhibited 58 proteins absent in the periodontitis samples. Importantly, exosomes from the periodontitis group were enriched with immune-related proteins, such as complement components and chemokine ligand 28, suggesting their active involvement in the immune response characteristic of the disease [55]. Interestingly, although EV size and morphology remained consistent across participants, the CD9 + subpopulation was more prevalent in those with periodontitis. A decrease in osterix mRNA and an increase in tumor necrosis factor-alpha (TNFα) were also observed, indicating their potential as diagnostic markers for periodontitis [56]. Furthermore, in the salivary EVs from periodontitis patients, higher levels of LPS + outer membrane vesicles, m^5^C methylation, and four periodontal pathogens were identified compared to healthy individuals. Notably, m^5^C hypermethylation in salivary EVs effectively distinguished periodontitis patients from both healthy and gingivitis groups [57].

**Fig. 3:**
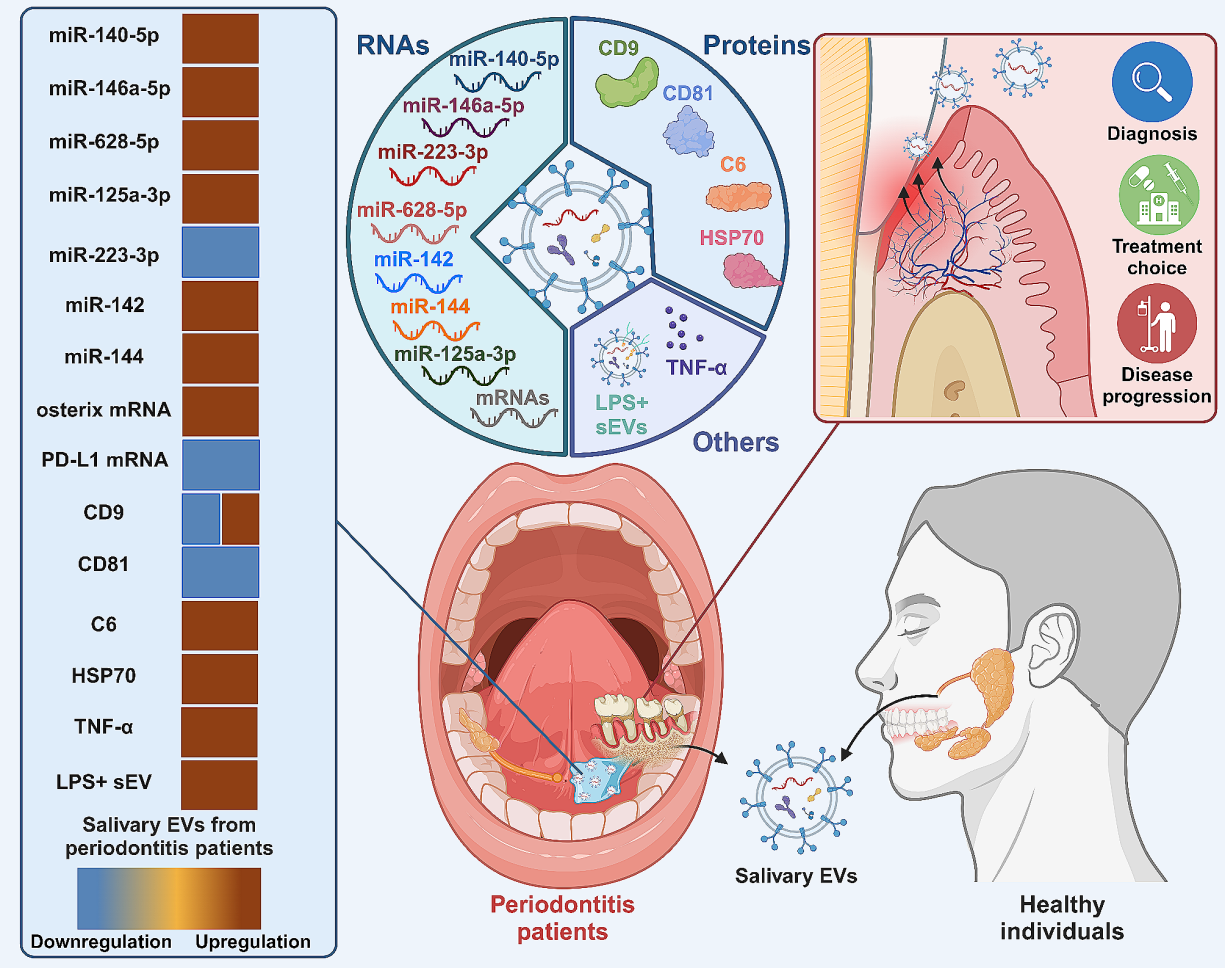
Salivary EVs in the diagnosis and monitoring of periodontitis. Notable differences in salivary exosomal miRNAs and proteins have been observed between healthy individuals and patients with periodontitis. Moreover, salivary EV profiles also provide robust biomarkers for assessing disease severity and responsiveness to therapy in periodontitis. This highlights the significant role of salivary EVs as valuable biomarkers in the diagnosis and ongoing monitoring of periodontitis

Beyond their diagnostic utility, salivary EV profiles also serve as robust biomarkers for disease severity and responsiveness to therapy in periodontitis. In patients with periodontitis, salivary samples demonstrated decreased levels of exosomal CD9 and CD81, when compared to healthy controls. Notably, these reduced levels correlated with clinical measures of periodontal disease severity [58]. Similarly, increased levels of PD-L1 mRNA were observed in salivary exosomes from periodontitis patients compared to controls, with variations in these levels indicating differences in disease severity. This suggests the potential utility of salivary PD-L1 mRNA as a diagnostic and prognostic marker [59]. Importantly, following initial periodontal therapy (IPT) for periodontitis, significant changes were observed in the composition of salivary exosomes. In particular, patients exhibiting increased periodontal inflammation post-IPT demonstrated elevated levels of C6 protein and miRNAs, including miR-142 and miR-144. Conversely, decreased or stable expressions of CD81 and TSG101 were associated with clinical improvements in periodontitis, such as reduced probing depth. Changes in HSP70 expression were consistent with persistent levels of periodontal inflammation [60] (Fig. 3).

#### Oral cancer

Oral cancer, defined as a malignant neoplasm found in the oral cavity, greatly benefits from early detection and treatment, which can significantly improve patient survival rates [61–63]. Compared to traditional biopsies and biochemical tests, saliva, being in direct contact with the site of oncogenic transformation, holds exceptional value for early diagnosis and disease monitoring. Cutting-edge diagnostic techniques have highlighted marked differences between salivary EVs in oral cancer patients and those in healthy individuals. For instance, salivary exosomes from oral cancer patients showed distinct fourier-transform infrared spectroscopy (FTIR) spectra compared to healthy individuals, especially at specific wavelengths linked to nucleic acids, lipids, and proteins. Machine learning models accurately differentiated between oral cancer and healthy samples, highlighting the potential of FTIR spectra of salivary exosomes as a non-invasive diagnostic tool for early oral cancer detection [64]. Moreover, using high-resolution atomic force microscope, salivary exosomes from oral cancer patients were found to be larger and displayed irregular morphologies compared to those from healthy individuals. Additionally, these cancer exosomes showed increased CD63 surface densities and unique multivesicular structures, suggesting that salivary exosomes could offer a new avenue for oral cancer diagnostics [65]. Furthermore, the surface enhancement Raman spectroscopy identified distinct variations in vibrational bands related to thiocyanate, proteins, and nucleic acids between salivary exosomes from cancer patients and healthy controls. The method demonstrated a relatively high discriminative power between the two groups [66].

The aberrant expression of salivary exosomal miRNAs holds potential for diagnosing and predicting the prognosis of oral cancer. Compared to healthy controls, miR-302b-3p and miR-517b-3p were exclusively present in the salivary exosomes from oral squamous cell carcinoma (OSCC) patients, whereas miR-512-3p and miR-412-3p showed a marked increase [67]. These findings highlight their potential as diagnostic markers for OSCC. Similarly, elevated levels of miR-24-3p were detected in salivary exosomes from OSCC patients, with a concurrent increase in OSCC tissues, suggesting an origin from tumor cells. Functionally, miR-24-3p facilitated malignant cell proliferation and interacted directly with the gene *PER1* [68]. Moreover, miR-1307-5p was significantly overexpressed in oral cancer tissues and their corresponding salivary exosomes, and its elevated expression correlated with unfavorable clinical outcome. Mechanistically, miR-1307-5p potentially drives cancer progression by targeting genes with tumor-suppressive functions [69]. Of the miRNAs identified in head and neck cancer patients’ plasma and saliva exosomes, 29 were tumor-specific. Ten of these displayed consistent levels in both biofluids, underscoring the potential to replace plasma with less-invasive saliva. Importantly, exosomal miRNA patterns were also found to be correlated with disease-free survival, HPV status, and disease stage [70].

**Fig. 4:**
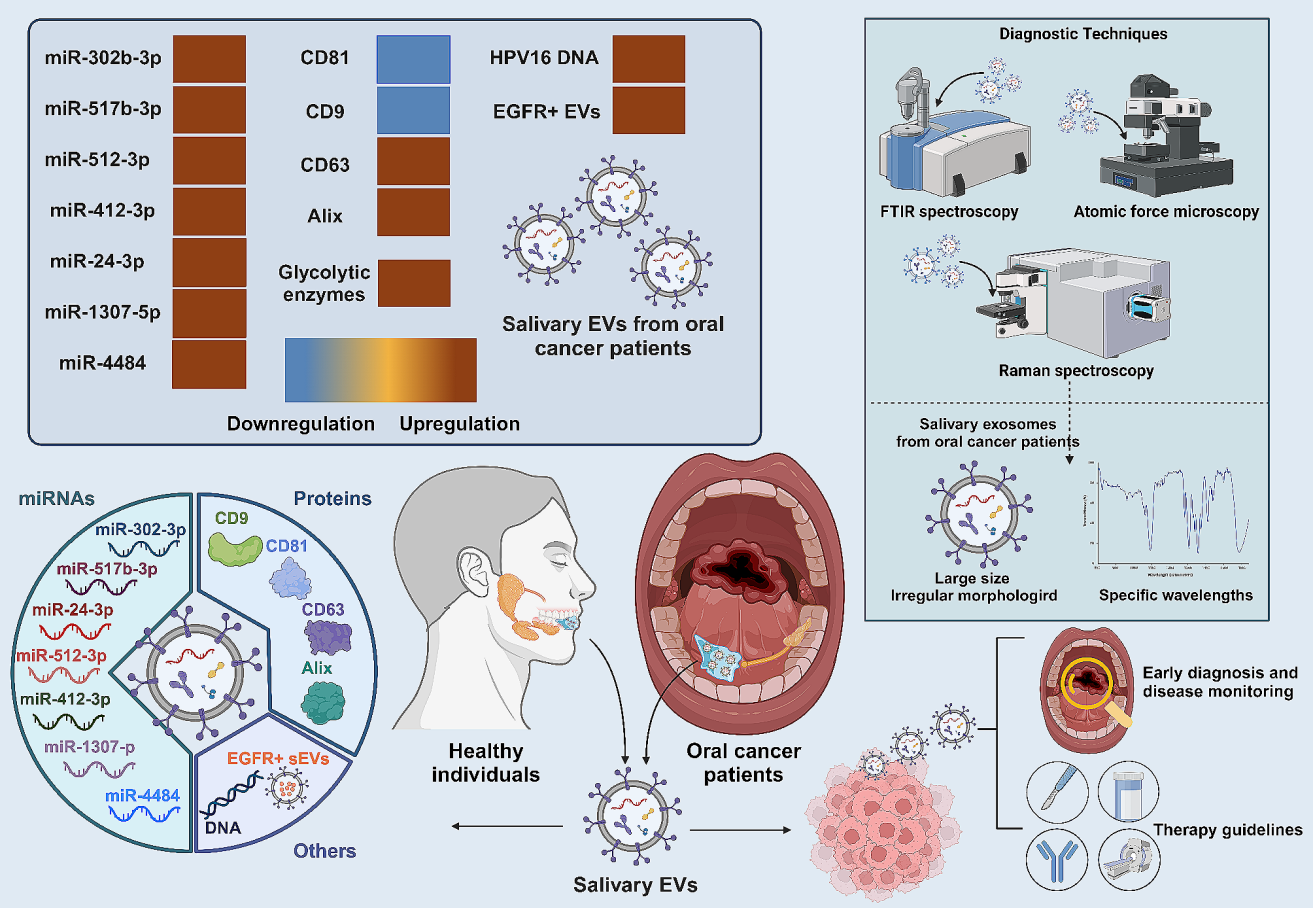
Salivary EVs as biomarkers for oral cancer. Salivary EVs are increasingly recognized as promising biomarkers for the accurate diagnosis and vigilant monitoring of oral cancer. Notably, distinct differences in the size and morphology of salivary EVs between individuals with oral cancer and healthy controls have been identified through detailed spectroscopic and morphological analyses. Moreover, oral cancer patients demonstrate aberrant expression profiles of microRNAs, proteins, and DNA in salivary EVs, highlighting their significant potential as biomarkers in oral oncology

Besides miRNAs, the protein and HPV DNA profiles in salivary exosomes from oral cancer patients also showed aberrant expression. Exosomes from the oral fluid of oral cancer patients exhibited both higher concentrations and larger sizes than those of healthy individuals. These exosomes had decreased levels of CD81 and CD9 but an elevated expression of CD63 [71]. In addition, quantitative proteome analysis of salivary small EVs from patients with varying stages of OSCC identified 365 differentially expressed proteins. The protein content within these vesicles displayed distinct functional signatures, underscoring their potential as novel predictive biomarkers for OSCC [72]. Moreover, exosomal Alix levels in serum and saliva were significantly higher in OSCC patients than in healthy control. In addition, serum exosomal Alix showed good performance for predicting the therapeutic outcome [73]. Similarly, the analysis, using liquid chromatography with tandem mass spectrometry and label-free protein quantification, identified that proteins related to inflammation, metal transport, and cellular growth are present in the proteome of salivary EVs from OSCC patients. Additionally, the proteomic data from these salivary EVs achieved a high accuracy rate of 90% in classifying OSCC [74]. Interestingly, HPV16 DNA was found in 80% of salivary exosomes from HPV-driven OPC patients. Additionally, these exosomes showed elevated levels of six key glycolytic enzymes, indicating a potential role in linking glucose metabolism with HPV-driven OPC [75]. Notably, salivary microvesicles (SMVs) are significantly increased in patients with OSCC compared to healthy individuals and those with oral ulcers. This elevation in SMVs correlates with more advanced lymph node involvement and higher clinical stages of OSCC. Furthermore, a lower ratio of apoptotic to non-apoptotic SMVs is associated with a higher pathological grade and poorer overall survival in OSCC patients [76]. Similarly, using a novel microfluidic approach, higher levels of EGFR^+^ EVs were found in the saliva of OSCC patients compared to healthy individuals, and the ratio of Annexin V^+^ EGFR^+^ EVs to Annexin V^−^ EGFR^+^ EVs correlated with the OSCC tumor T stage [77]. This technique offers a promising tool for real-time monitoring of OSCC progression and assessing tumor stage (Fig. 4).

Critically, investigating the changes in salivary EV molecular profiles following a range of treatments, such as surgery, chemoradiotherapy, targeted therapy, and immunotherapy, is essential for monitoring therapeutic outcomes in oral cancer. Salivary EVs, carrying diverse molecular information, could offer real-time insights into the tumor environment and how the body responds to treatment. This approach has the potential to provide a non-invasive method for evaluating treatment efficacy and aiding in the development of personalized therapeutic strategies.

### Other oral diseases

In addition to periodontitis and oral cancer, altered salivary exosome profiles have been observed in oral lichen planus (OLP), a mucosal disease with potential for malignant transformation. The marked increase of miR-4484 in OLP patient salivary exosomes suggests its utility as a specific biomarker for early detection, enabling timely intervention and possibly preventing cancerous progression. Similarly, a rise in EVs in peri-implant crevicular fluid was noted in peri-implantitis cases [79], indicating that EV concentration monitoring in such fluids or saliva could offer a non-invasive diagnostic tool for this condition. Furthermore, distinct serum exosomal miR-671-5p, miR-16-5p, and miR-150-3p expressions were significantly altered in patients with hand, foot, and mouth disease (HFMD) compared to healthy individuals. Given the non-invasive nature of salivary diagnostics, further research into salivary exosome alterations may provide insights into HFMD’s diagnosis and progression monitoring [80]. Unfortunately, the exploration of salivary EVs in the context of other oral diseases, such as oral leukoplakia, and early childhood caries is currently limited. Focusing research on salivary exosomes in these areas may significantly enhance disease monitoring. By identifying specific biomarkers within these EVs, it may be possible to detect early stages of disease development, allowing for more timely and effective interventions. The summary of salivary exosomal biomarkers for oral diseases in the review is provided in Table 2.

**Table 2:** Salivary EVs as biomarkers for oral disease diagnosis and monitoring

Disease	Salivary EV markers	Alteration	Clinical significance	Ref.
Periodontitis	miR-140-5p, miR-146a-5p, miR-628-5p	Increased	Diagnostic markers for periodontitis	[52]
	miR-223-3p	Decreased	Diagnostic markers for periodontitis	[53]
	miR-125a-3p	Increased	Diagnostic markers, correlates with pocket depth	[54]
	Immune-related proteins	Increased	Diagnostic markers, involvement in immune responses	[55]
	CD9, *osterix*, *TNFα*	Increased: CD9, *TNFα* Decreased: *osterix*	Diagnostic markers for periodontitis	[56]
	LPS^+^ outer membrane vesicles, m^5^C methylation, pathogens	Increased	Diagnostic markers for periodontitis	[57]
	CD9, CD81	Decreased	Correlates with periodontal disease severity	[58]
	*PD-L1* mRNA	Increased	Correlates with periodontal disease severity	[59]
	C6, miR-142, miR-144 CD81, TSG101	Increased: C6, miR-142, miR-144 Decreased or unchanged: CD81, TSG101	Potential biomarkers for monitoring therapeutic efficacy	[60]
Oral cancer	Size, morphology, contents	Altered	Diagnostic markers for oral cancer	[64–66, 71]
	miR-302b-3p, miR-517b-3p, miR-512-3p, miR-412-3p	Increased	Diagnostic markers for oral cancer	[67]
	miR-24-3p	Increased	Diagnostic marker for oral cancer	[68]
	miR-1307-5p	Increased	Diagnostic and prognostic markers for oral cancer	[69]
	CD81, CD9, CD63	Increased: CD63 Decreased: CD81, CD9	Diagnostic marker for oral cancer	[71]
	the proteomic pattern	Altered	Diagnostic marker for oral cancer	[71, 74]
	Alix	Increased	Diagnostic and prognostic markers for oral cancer	[73]
	HPV DNA, glycolytic enzymes	Increased	Diagnostic marker for oral cancer	[75]
	salivary microvesicles	Increased	Diagnostic and prognostic markers for oral cancer	[76]
	EGFR^+^ EVs	Increased	Diagnostic and prognostic markers for oral cancer	[77]
Oral lichen planus	miR-4484	Increased	Biomarker for monitoring malignant transformation	[78]

### Salivary EVs as biomarkers for systemic disease diagnosis and monitoring

Salivary EVs are increasingly recognized for their role in systemic disease surveillance due to their ability to harbor biomarkers from the bloodstream. The vascularization of salivary glands allows molecules from systemic circulation to be captured and concentrated in salivary EVs. Consequently, these vesicles might carry and convey complex information about systemic pathophysiological states, including metabolic disorders, autoimmune diseases, and malignancies. For instance, using a human lung cancer mouse model, it was found that exosome-like microvesicles transport tumor-specific proteins and mRNAs, such as human GAPDH mRNA, from blood to saliva. This confirms their potential as indicators of tumor progression that can be detected through saliva [81]. Isolation and analysis of salivary EVs allow for a minimally invasive, real-time assessment of systemic health, which is valuable for early detection of systemic diseases, monitoring their progression, and evaluating treatment efficacy.

#### Primary Sjögren’s syndrome (pSS)

pSS is an autoimmune disorder characterized primarily by chronic inflammation of the salivary and lacrimal glands, leading to dry mouth and dry eyes. The syndrome can also be associated with systemic features and has the potential to affect various organs and cause a wide spectrum of symptoms [82, 83]. In pSS, both saliva and its EVs display an upregulation of proteins crucial for innate immunity and cell signaling. Specifically, saliva EVs contain biomarkers indicative of innate immune activation and adipocyte differentiation [84]. Similarly, using a SWATH-MS approach, Finamore et al. profiled the sub-proteome of extracellular vesicle-enriched saliva in pSS patients and identified proteins differentially expressed between pSS and healthy control EV samples. A significant number of proteins, primarily associated with the immune response, were upregulated in pSS patient saliva EVs [85]. Interestingly, microarray technology revealed distinct RNA profiles in salivary EVs from pSS patients. Notably, levels of tRNA-Ile-AAT-2-1 were found to be reduced by half. These findings collectively position salivary EVs as valuable for discovering new pSS biomarkers, which could assist in diagnosis, patient stratification, and treatment monitoring [86]. It should be noted that salivary EVs hold promise for distinguishing pSS from other conditions. While non-SS sicca subjects exhibited mild glandular inflammation, pSS patients showed pronounced proinflammatory pathways in tear fluid. Importantly, immune-regulatory proteins were identified in EVs from both tear fluid and saliva in pSS patients, underscoring the potential of these EVs as dual, non-invasive biomarkers for differentiating between pSS and non-SS sicca symptoms [87].

#### Non-oral neoplasms

Beyond their utility in oral cancer, salivary EVs demonstrate significant potential for early detection and monitoring of various other cancer types. For instance, salivary exosomes enriched with certain small RNAs, including tRNA-GlyGCC-5 and an uncharacterized small RNA, provide a powerful bi-signature for detecting esophageal squamous cell carcinoma (ESCC). This bi-signature demonstrates high sensitivity and specificity in distinguishing ESCC patients from non-cancer individuals and has been linked to significant differences in overall and progression-free survival, indicating its potential as a prognostic biomarker for ESCC [88]. Similarly, in ESCC, salivary exosomal G-NchiRNA levels correspond with tumor burden and decrease after tumor resection. This biomarker also effectively evaluates chemoradiation response and predicts disease progression, potentially earlier than imaging. Changes in salivary exosomal G-NchiRNA levels can prognosticate progression-free survival, highlighting its utility for noninvasive monitoring of ESCC treatment and recurrence [89].

Utilizing a digital PCR chip with microfluidic technology, this approach effectively distinguishes lung cancer patients from healthy controls by analyzing salivary EVs, demonstrating high accuracy and superior performance to traditional qPCR, even in samples with low concentrations [90]. Additionally, label-free quantification revealed 319 proteins in saliva exosomes and 994 in serum exosomes. A comparative proteome analysis between healthy individuals and lung cancer patients identified 11 protein candidates common to both fluids in lung cancer patients, indicating that circulating exosomes contain cancer-related proteins and hold promise for the early detection and diagnosis of lung cancer [91]. Analysis of salivary exosomes in lung cancer patients identified 15 differentially expressed miRNAs, including eight upregulated and seven downregulated. These deregulated miRNAs are linked to cancer-associated pathways, underscoring their potential as biomarkers for lung cancer diagnosis [92].

Although no significant differences were found in the size and concentration of saliva EVs between pre- and postoperative samples from glioblastoma multiforme (GBM) patients, preoperative samples showed a higher protein content and identified specific proteins associated with poor outcomes. These findings suggest that salivary EVs could offer a non-invasive approach to GBM prognosis, warranting further research in larger patient cohorts [93]. In a study targeting early detection of pancreatobiliary tract cancer, miR-1246 and miR-4644 were significantly elevated in salivary exosomes of cancer patients compared to healthy controls. The combination of these miRNAs showed high diagnostic accuracy, suggesting their promise as non-invasive biomarkers for this hard-to-diagnose cancer [94].

#### Parkinson’s disease (PD)

PD is a progressive neurodegenerative disorder characterized by the loss of dopamine-producing neurons in the substantia nigra, a region of the brain critical for regulating movement [95]. Emerging research indicates that neurodegenerative conditions such as PD could alter the composition of salivary exosomes. Since exosomes are capable of traversing the blood-brain barrier and play a key role in intercellular communication, they might transport specific neural biomarkers into the saliva, thereby providing a non-invasive diagnostic window into brain health. For instance, the levels of phosphorylated α-synuclein in salivary exosomes are notably greater in patients with PD. Additionally, there is a greater presence of neuron-derived salivary exosomes in PD patients [96]. This suggests that PD patients may have an elevated release of exosomes from neuronal cells within the salivary glands. Similarly, saliva samples from 74 PD patients and 60 healthy controls showed that levels of oligomeric α-synuclein, and its ratio to total α-synuclein in EVs, were higher in PD patients. However, no significant correlation was found between these markers and disease severity [97]. Interestingly, Rastogi et al. presents a novel approach for PD diagnosis using fluorescence-tagged EVs in saliva. It was found that EV concentrations were significantly higher in PD patients compared to healthy controls, and this increase was also notable in prodromal PD cases. The total α-synuclein levels within these EVs further confirmed their higher presence in PD [37]. Furthermore, lower concentrations of total α-synuclein in salivary EVs were observed in multiple system atrophy-parkinsonism (MSA-P) than in PD. In addition, distinct external anal sphincter electromyography features also distinguished MSA-P and PD. This combined method offered high sensitivity and specificity, suggesting its significant potential for accurate differential diagnosis [98].

#### Traumatic brain injury (TBI)

Substances traverse the blood-brain barrier (BBB) through either paracellular or transcellular pathways. In the paracellular route, the passage of exosomes is passively regulated and constrained by the size of intercellular pores. In contrast, the transcellular pathway, involving active transport across cellular membranes, emerges as a more effective mechanism for shuttling exosomes across the BBB [99]. This pathway within BBB endothelial cells is characterized by a bidirectional process: it not only involves endocytosis at the apical membrane, intracellular transport, and release at the basolateral side but also supports the reciprocal movement, permitting the exchange of substances between the brain and the bloodstream in both directions. Notably, the efficiency of exosome transit across the BBB is also influenced by the barrier’s integrity and the physiological state of brain tissues, as well as the exosomes’ physical properties [100]. This bidirectional transport facilitates the movement of brain-originated exosomes to other organ systems. TBI is an alteration in brain function or other evidence of brain pathology that result from external forces [101]. TBI may influence the molecular composition of salivary EVs. Following a traumatic event, the disrupted BBB and inflammatory responses may lead to an altered profile of biomarkers released from brain cells, which can be reflected in the saliva. By comparing pre- and post-fight salivary EV gene expression, significant changes were observed, particularly following high-impact injuries or prolonged bouts. The findings suggest that salivary EVs could be a potential biomarker for TBI severity and assist in unraveling the pathophysiological mechanisms of head injuries in combat sports [102]. Similarly, analysis of salivary EVs using the TaqMan Human Inflammation array revealed upregulation of inflammation-related genes in patients with TBI [103]. Interestingly, real-time PCR analysis of salivary EVs targeting Alzheimer’s-related genes revealed 57 upregulated genes in emergency department TBI patients and 56 in concussion clinic patients, with CDC2, CSNK1A1, and CTSD elevated in both groups. This suggests salivary EVs could be a non-invasive biomarker for mild TBI, and also indicates a possible connection between mTBI and Alzheimer’s gene expression [104].

#### Other systemic conditions

**Fig. 5:**
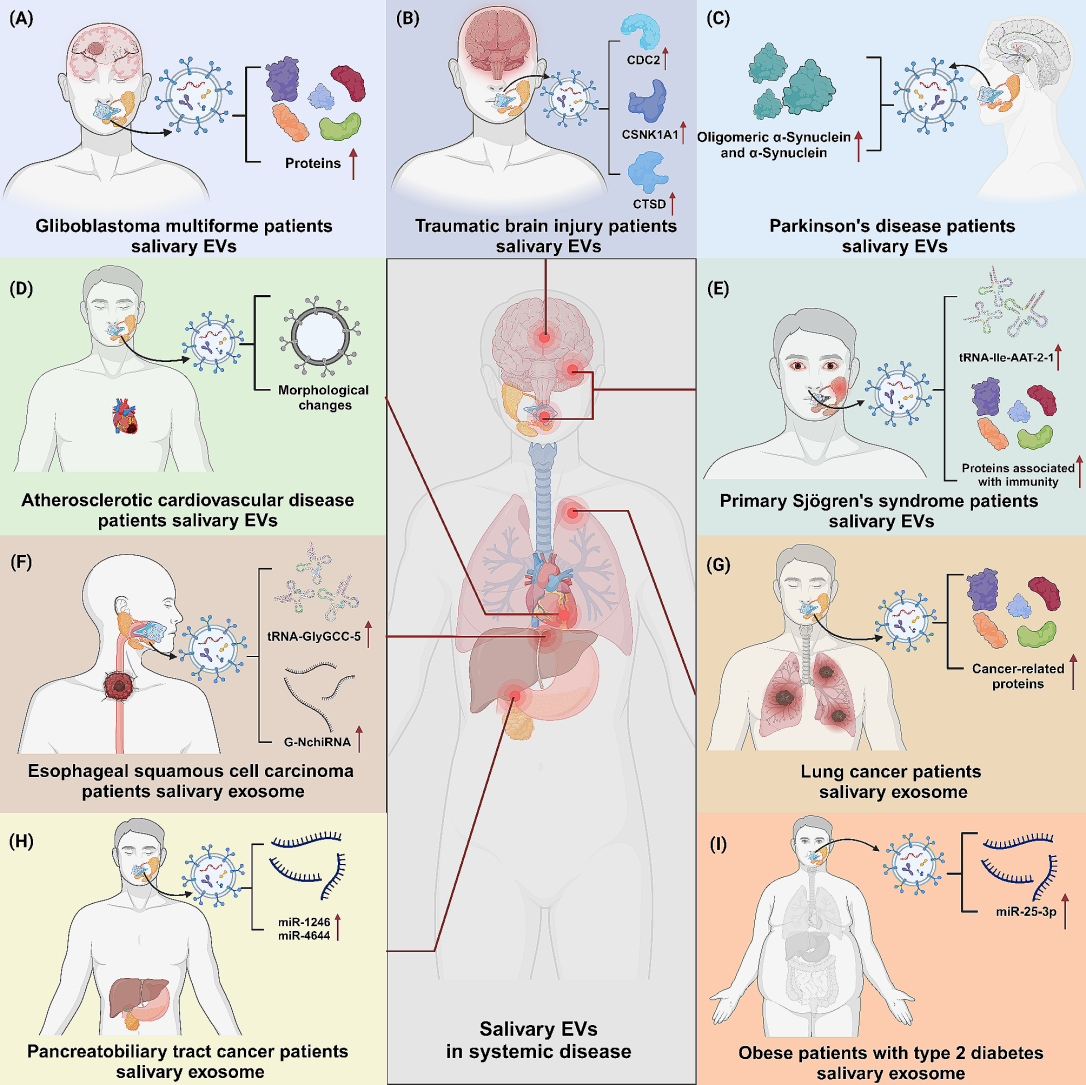
Salivary EVs are emerging as promising biomarkers for the diagnosis and monitoring of various systemic conditions. (**A**) In GBM patients, salivary exosomes show a notable increase in protein content. (**B**) Post-TBI, salivary EVs exhibit elevated expression of CDC2, CSNK1A1, and CTSD. (**C**) In PD patients, there’s an enhanced presence of α-synuclein, especially oligomeric forms, in salivary EVs. (**D**) EV morphology alterations are observable in AsCVD patients. (**E**) pSS patients exhibit increased levels of both tRNA and proteins in their salivary EVs. (**F-H**) Significantly, in ESCC, lung cancer, and pancreatobiliary tract cancer patients, there’s a marked elevation in tRNA, miRNA, and protein levels in salivary EVs. (**I**) In obese type 2 diabetes patients, salivary exosomal miR-25-3p is significantly elevated

Salivary EVs also demonstrate significant potential in the diagnosis and monitoring of various systemic conditions. For instance, notable morphological changes were observed in small EVs from various biofluids, including plasma, saliva and urine, in patients with atherosclerotic cardiovascular disease (AsCVD) compared to healthy individuals [105]. In addition, salivary exosomal miR-25-3p is significantly enriched in obese patients with type 2 diabetes and is implicated in the progression of periodontitis. This miRNA targets CD69 mRNA, influencing γδ T cell activation and inflammatory responses. In mouse models, inhibiting miR-25-3p curtailed inflammation and mitigated periodontal bone loss, suggesting a potential therapeutic avenue for diabetes-associated periodontitis through modulating local immune responses [106]. Moreover, saliva-derived EVs from asthma patients can be isolated effectively for large-scale studies and highlights the potential of these EVs as novel biomarkers for asthma [107]. Furthermore, a multiomics analysis of saliva exosomes from medical residents, conducted before and after a 12-hour work shift, revealed significant variations in specific proteins and miRNAs. These changes, particularly in phosphoglycerate kinase 1, correlated with mood states like fatigue [108]. Interestingly, salivary exosomes from climacteric females had higher total protein content and larger size, whereas adolescent female derived salivary exosomes displayed a greater variety of proteins. This age-related variation in salivary exosomes’ protein composition offers valuable insights for potential physiological changes in these distinct female age groups [109]. Similarly, a number of salivary exosomal miRNAs exhibited aberrant expression between older and younger individuals, among which miR-24-3p was validated to be significantly elevated in the aging group [110] (Fig. 5). The summary of salivary exosomal biomarkers for systemic diseases in the review is provided in Table 3.

**Table 3:** Salivary EVs as biomarkers for diagnosis and monitoring of systemic diseases

Disease	Salivary EV markers	Alteration	Clinical significance	Ref.
pSS	proteins associated innate immunity	Increased	Involvement in pathogenesis of pSS	[84, 85]
	RNA profiles, tRNA-Ile-AAT-2-1	Alter, decreased	Diagnostic markers for pSS	[86]
	immune-regulatory proteins	Increased	Differentiating pSS from non-SS sicca	[87]
Non-oral neoplasms	tRNA-GlyGCC-5	Increased	Diagnostic marker for ESCC	[88]
	G-NchiRNA	Decreased	Prognostic marker for ESCC	[89]
	the proteomic pattern	Altered	Diagnostic markers for lung cancer	[91]
	aldolase A, 14-3-3 protein ε, enoyl CoA hydratase 1, and transmembrane protease serine 11B	Increased	Prognostic marker for GBM	[93]
	miR-1246 and miR-4644	Increased	Diagnostic marker for pancreatobiliary tract cancer	[94]
Parkinson’s disease	α-synuclein	Increased	Diagnostic markers for Parkinson’s disease	[96, 97]
	EV concentration, α-synuclein	Increased	Diagnostic markers for Parkinson’s disease	[37]
	α-synuclein	Increased	Differentiating Parkinson’s disease from MSA-P	[98]
TBI	gene expression pattern	Altered	Diagnostic markers for TBI	[102]
	inflammation-related genes	Increased	Diagnostic marker for TBI	[103]
	CDC2, CSNK1A1, CTSD	Increased	Diagnostic marker for mild TBI	[104]
Other systemic conditions	morphology	Altered	Involved in atherosclerotic cardiovascular disease	[105]
	miR-25-3p	Increased	Correlated with progression of diabetes-associated PD	[106]
	PGK1	Increased	Correlated with mood states	[108]
	morphology, protein content	Altered	Correlated with aging	[109]
	miR-24-3p	Increased	Correlated with aging	[110]

### The therapeutic potential of salivary EVs

In addition to their diagnostic and prognostic value, salivary EVs also possess therapeutic potential. This potential lies in their intrinsic physiological functions and the complex molecular cargo they carry. Acting as natural carriers, these vesicles facilitate intercellular communication between cells. In the therapeutic context, salivary EVs could be utilized to deliver specific therapeutic molecules to targeted tissues or cells. Their ability to encapsulate and protect RNA and proteins from enzymatic degradation makes them particularly suitable for transporting genetic materials or proteins that can influence disease pathways. For instance, salivary EVs can inhibit Zika virus (ZIKV) infectivity in physiological concentrations by blocking viral attachment to target cells. This antiviral activity of salivary EVs, effective against ZIKV, is not impacted by heat treatment, suggesting that the inhibitory factor is non-proteinaceous [111]. Additionally, human salivary EVs exhibit coagulant tissue factor (TF) and CD24, which facilitate hemostasis by adhering to activated platelets through P-selectin interactions. Despite the absence of PSGL-1, the critical co-localization of TF and CD24 on these EVs is essential for their clotting function, as demonstrated by the loss of coagulant activity when CD24-exposing EVs are removed. This indicates that salivary EVs could play a significant role in the body’s innate response to oral injuries [112]. Moreover, salivary exosomes promote the proliferation, migration, and angiogenic activity of human umbilical vein endothelial cells in vitro and expedite cutaneous wound repair in vivo. A key component of salivary exosomes, UBE2O, mirrors the pro-angiogenic effects of the exosomes by decreasing SMAD6 levels and activating BMP2. These findings point to salivary exosomes and UBE2O as potential therapeutic agents for promoting angiogenesis in wound healing [113]. Furthermore, administering salivary exosomes to diabetic rats led to lowered blood glucose and improved gland function, as evidenced by reduced water intake, serum nitric oxide, and increased salivary flow and amylase levels. Histological improvements and reduced expression of NFκB/p65 and TNFα also supported these effects. These results suggest salivary exosomes as a potential cell-free therapy for DM-related xerostomia and gland dysfunction [114]. Interestingly, salivary exosomes from mice with pancreatic ductal adenocarcinoma can suppress natural killer (NK) cell activity. This suppression of NK cell cytotoxicity is not observed when pancreatic tumors are engineered to reduce exosome secretion, highlighting the potential of tumor exosomes to alter immune function at distant sites [115]. Furthermore, rat salivary nanovesicles contains nucleotidases such as NTPDase1, -2, -3, and ecto-5’-nucleotidase, capable of hydrolyzing ATP. These enzymes within salivary EVs are pivotal for modulating ATP and adenosine levels, significantly influencing purinergic signaling in oral tissues [116].

Beyond utilizing physiological salivary EVs for therapeutic potential, the inherent biocompatibility and low immunogenicity of salivary EVs make them excellent candidates for drug delivery vehicles. By bioengineering these vesicles to present specific surface ligands, it is possible to achieve targeted delivery of therapeutic agents to pathologically affected cells or tissues. This precise delivery mechanism has the potential to enhance the efficacy of pharmacological interventions while concurrently mitigating systemic adverse effects. This strategy has wide-ranging potential across various medical fields. In oncology, for example, salivary EVs can be engineered to deliver chemotherapy agents directly to cancer cells, localizing treatment effects and reducing harm to healthy tissue.

**Fig. 6:**
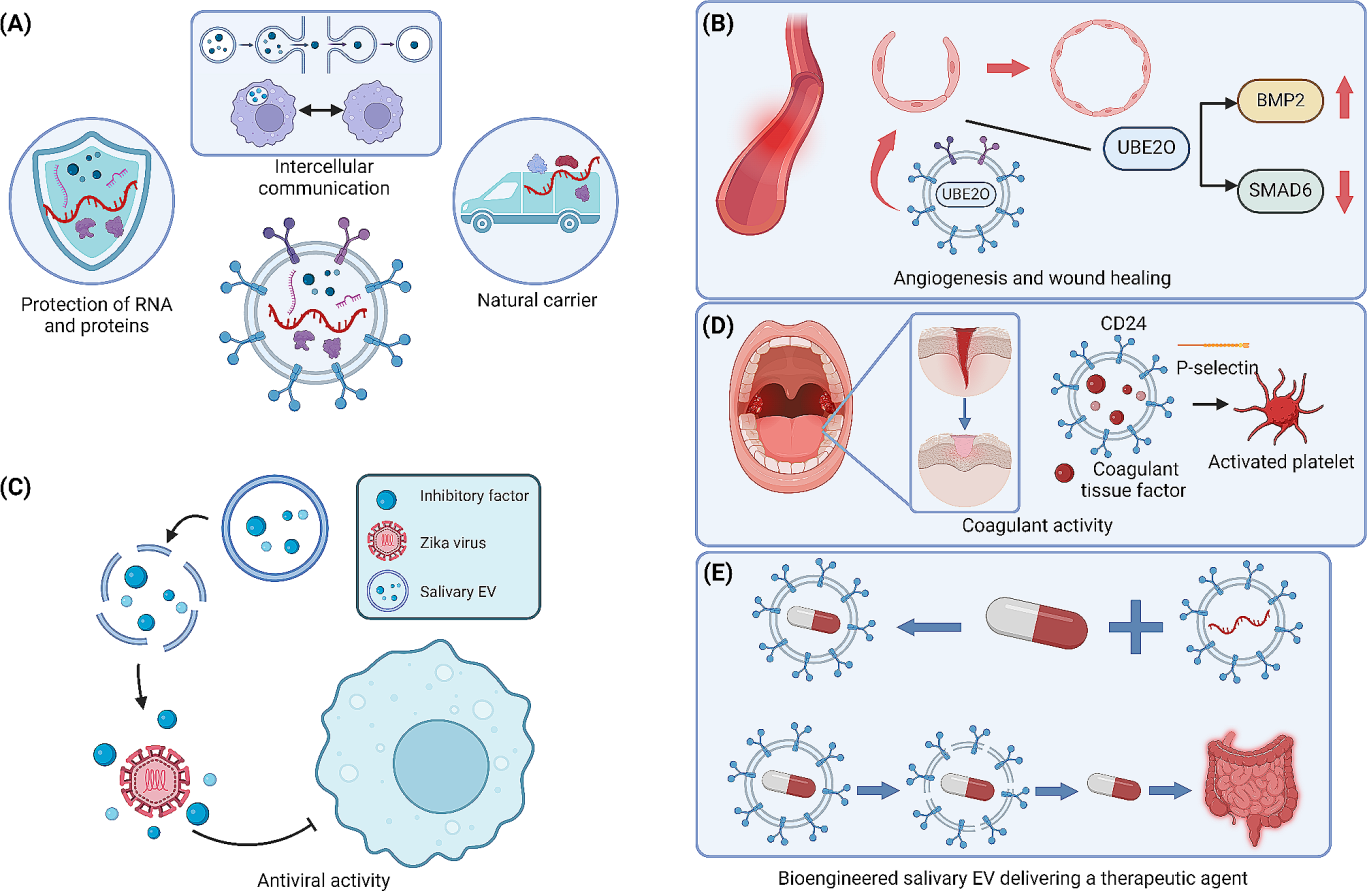
The therapeutic potential of salivary EVs. (**A**) Beyond their roles in diagnostics and prognosis, salivary EVs exhibit significant therapeutic potential. This arises from their inherent physiological functions and the complex array of molecular cargo they transport. (**B**) Salivary EVs are pivotal in promoting angiogenesis and enhancing wound healing processes. (**C**) Notably, salivary EVs have shown effective antiviral activity, especially against ZIKV infections, attributed to nonprotein inhibitory factors. (**D**) Containing coagulant tissue factor and CD24, salivary EVs contribute to hemostasis by binding to activated platelets via P-selectin interactions. (**E**) These distinctive capabilities render salivary EVs as optimal vectors for the delivery of specific therapeutic molecules to targeted tissues, thus advancing the prospects of precision medicine

For autoimmune diseases, modified EVs could transport immunomodulatory drugs directly to overactive immune cells, offering a more targeted approach than traditional systemic therapies. In the field of genetic therapy, salivary EVs could be designed to transport gene-modifying elements like siRNAs or mRNAs to specific cells, ushering in a new era of gene therapy. This is especially promising for treating genetic disorders that require precise gene alteration or regulation. Thus, the use of bioengineered salivary EVs as therapeutic vectors represents an advanced and dynamic area of medical research, providing innovative methods for precise and targeted treatment modalities (Fig. 6).

### Current challenge and future prospects

The utilization of salivary EVs as non-invasive biomarkers presents an innovative approach in the realm of early disease detection and prognosis prediction. Nevertheless, several obstacles still exist in bridging the gap between fundamental research and practical clinical application. Upon reviewing current studies on salivary EVs for disease diagnosis and monitoring, several limitations become evident. A primary concern is the small size of study cohorts, which increases the risk of false positives and challenges in replicating results, thereby questioning the reliability and applicability of the findings. For instance, contradictory findings were observed regarding the levels of salivary exosomal CD9 in periodontitis [58, 117]. To address this, larger and more diverse sample pools are essential to enhance the generalizability of research outcomes across varied patient demographics. In addition, while the detection of α-synuclein in PD patients’ salivary EVs is established, the robustness of other biomarkers related to disease progression requires further verification. This necessitates comprehensive, independent research to confirm these biomarkers’ clinical relevance, specificity, and reliability. Furthermore, the absence of large-scale clinical trials utilizing salivary biomarkers represents a notable gap in the field. Conducting such trials is vital to evaluate the real-world clinical utility of these biomarkers, understanding their practical limitations and potential.

Despite advancements in EV isolation techniques based on physical and biochemical characteristics, and the advent of technologies like microfluidics, a universally accepted protocol for efficient, high-throughput, and minimally invasive isolation of salivary EVs has yet to be established. The primary obstacles include the low sensitivity in differentiating EV subsets and the complex and heterogeneous nature of biological samples [118]. Innovative and sensitive methodologies are therefore essential for the isolation of EVs from saliva, alongside robust validation of their contents as disease biomarkers. In the field of exosome manufacturing for therapeutic and diagnostic purposes, several key challenges are currently being faced. Scalability is a major concern, as existing methods for isolating and purifying exosomes are not adequately equipped for large-scale production. The development of three-dimensional stirred-tank bioreactors is imperative to yield exosomes in quantities sufficient for therapeutic applications. Additionally, the safety and purity of exosome preparations are critical. Ensuring that these preparations are free from contaminants such as proteins, or nucleic acids is crucial for their effectiveness and safety in therapeutic use. Additionally, it is important to prevent contamination with harmful substances during the manufacturing process. There is a pressing need for more precise and scalable purification techniques to produce exosomes of high purity, especially for human use in clinical trials during drug development phases [119]. Moreover, standardization and quality control also present significant hurdles. Establishing consistent and standardized protocols for exosome manufacturing is essential to ensure reproducibility and reliability in clinical applications.

In addition, in the context of tumor diagnostics, a critical challenge arises in distinguishing and enriching tumor-derived EVs within saliva, which may be confounded by EVs originating from non-tumorigenic cells. While immune affinity-based enrichment methods utilizing cell-specific surface proteins have shown promise, their efficacy is often compromised by the proteolytic cleavage of surface markers [120]. Therefore, ongoing research endeavors are directed towards the identification and validation of stable, specific markers for the efficient enrichment of tumor-derived EVs, a step crucial for the clinical application of salivary EVs in cancer diagnostics.

Moreover, a deeper understanding of the relationship between diseased organs involved in systemic diseases and the salivary glands is essential. This understanding is critical for comprehending how systemic conditions, such as autoimmune disorders or cancers, might influence the composition and function of salivary EVs. This knowledge is pivotal not only for developing more sophisticated diagnostic tools that utilize salivary EVs but also for potentially creating therapeutic strategies targeting these inter-organ communications.

Furthermore, fully deciphering the molecular cargo of salivary EVs has profound implications for therapeutic interventions. For instance, dipeptidyl peptidase IV within salivary EVs retains its activity following digestion, suggesting a potential role for saliva-derived EVs in delivering active molecules to the gastrointestinal tract and aiding in maintaining its homeostasis [34]. Therefore, in conditions like inflammatory bowel disease or intestinal infections, where the mucosal barrier is compromised, delivering specific anti-inflammatory agents or growth factors via salivary EVs could promote mucosal healing and restore barrier function. Additionally, in metabolic disorders like diabetes, where dysregulation of gut hormones impacts glucose metabolism, salivary EVs could be engineered to deliver molecules that modulate these hormones or directly affect glucose absorption and insulin sensitivity.

Interestingly, high levels of anti-immune subgenomic flaviviral RNA (sfRNA) in dengue-infected mosquito saliva, encapsulated in EVs, are key in enhancing virus infectivity and suppressing human immune responses. Depleting sfRNA in these salivary EVs could be a strategic approach for controlling dengue transmission, highlighting a potential avenue for disease prevention [121]. This concept of specifically targeting salivary EVs could be broadened to encompass various diseases where salivary EVs contribute to pathogenesis, such as in the spread of oncogenic viruses or the transportation of disease-promoting molecules. In the realm of autoimmune diseases, for example, removing harmful autoantigens carried by salivary EVs might mitigate symptoms and impede disease progression. The development of targeted interventions, potentially leveraging molecular editing techniques like CRISPR-Cas9 or specific neutralizing agents, is essential for this approach. This strategy of selectively depleting harmful components from salivary EVs holds significant promise, offering a targeted and potentially transformative addition to current therapeutic modalities.

## Conclusions

In summary, salivary EVs mark a significant advancement in medical diagnostics and treatment strategies. These tiny but powerful entities have shown remarkable potential in early disease detection and monitoring. Their ability to act as non-invasive biomarkers simplifies the diagnostic process, making it more patient-friendly. Furthermore, the prospect of using these vesicles in targeted therapies opens up new possibilities for personalized medicine. However, the path forward involves overcoming multiple challenges to ensure the reliability and effectiveness of their use in clinical applications. As research in this field advances, salivary EVs are poised to become an integral part of future healthcare, transforming our approach to disease management and patient care.

## Data Availability

No datasets were generated or analysed during the current study.

## Abbreviations

EVs: extracellular vesicles
OSCC: oral squamous cell carcinoma
sfRNA: subgenomic flaviviral RNA
NK cell: natural killer cell
ZIKV: Zika virus
TBI: traumatic brain injury
pSS: primary Sjögren’s syndrome
ESCC: esophageal squamous cell carcinoma
GBM: glioblastoma multiforme
miRNAs: microRNAs
TNFα: tumor necrosis factor-alpha
SEC: size exclusion chromatography
PEG: polyethylene glycol
FTIR: fourier-transform infrared spectroscopy
MVBs: multivesicular bodies
MSA-P: multiple system atrophy-parkinsonism
PEG: polyethylene glycol
DEP: dielectrophoretic
DLD: deterministic lateral displacement
OLP: oral lichen planus
HFMD: hand, foot, and mouth disease
BBB: blood-brain barrier
